# Supplementary figures and images for: The Syncytial Drosophila Embryo as a Mechanically Excitable Medium
Source: PLoS One. 2013 Oct 30;8(10):e77216. doi: 10.1371/journal.pone.0077216 (PMC3813724; doi:10.1371/journal.pone.0077216)

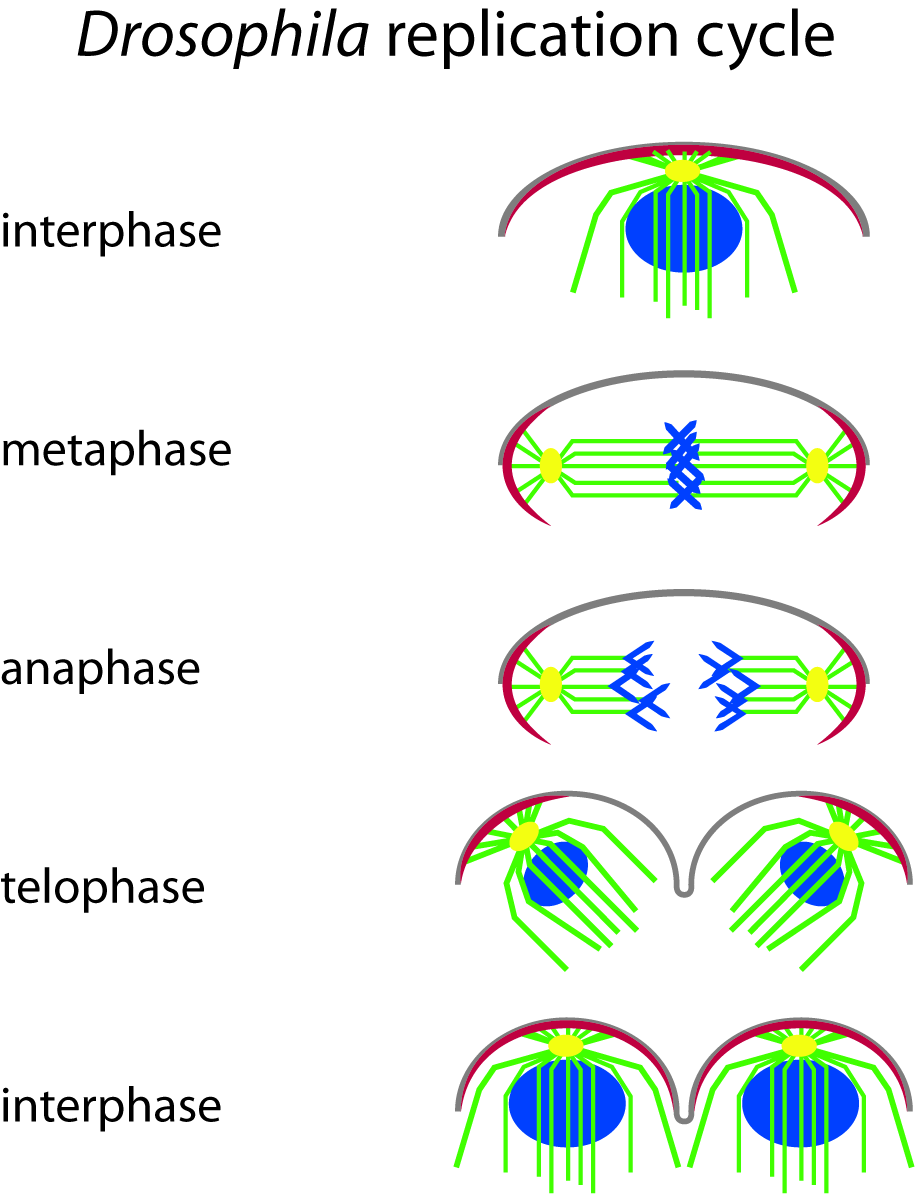

Supplement: Figure S1 — Illustration showing the four stages of the Drosophila embryo replication cycle that can be detected from our movies: interphase (DNA replication), metaphase (condensation of chromosomes in the nuclear midplane), anaphase (division of the nucleus in two daughter nuclei) and telophase (separation of daughter nuclei). The plasma membrane is shown in gray, the actin cap (made of actin filaments) in red, the microtubules in green, the centrosomes in yellow, and the DNA/chromosomes in blue. (TIFF) [file pone.0077216.s001.tiff]

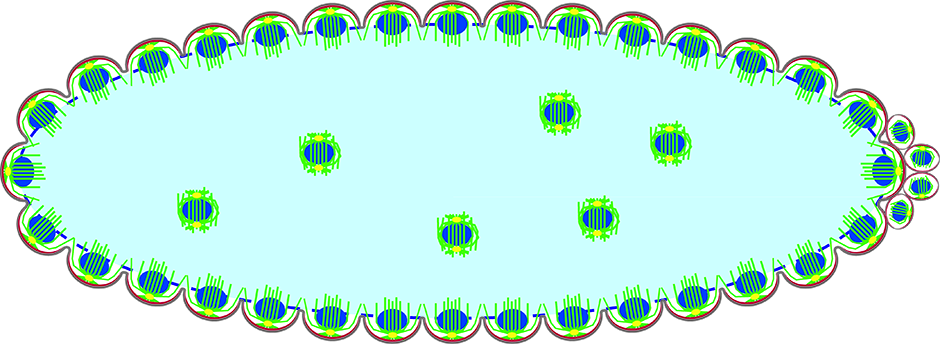

Supplement: Figure S2 — Sketch of a cross-section through a Drosophila embryo valid for stages 9–13. Most nuclei are located at the surface of the embryo. The nuclei are pushed outwards into the plasma membrane (gray), resulting in the formation of somatic buds. Each nucleus is enclosed in a microtubule basket (green) and contained in an individual actin cap (red), which gets disassembled after mitosis and re-assembled during interphase. DNA/chromosomes are shown in blue and centrosomes in yellow. The yolk (light blue) is a viscoelastic fluid containing water, cytoskeletal elements and necessary building blocks for the nuclei. The yolk is bounded by an actin cortex over which the nuclei can move. Also shown in this sketch are the small number of nuclei that reside inside the yolk, and the also small number of somatic cells that already form in cycle 10 at the posterior end (the pole cells that divide out of sync with the rest of the embryo). See Foe and Alberts [1] for sketches for each of the first 14 cycles and Schejter and Wieschaus [4] for a review on the cytoskeletal elements in the early embryo. (TIFF) [file pone.0077216.s002.tiff]

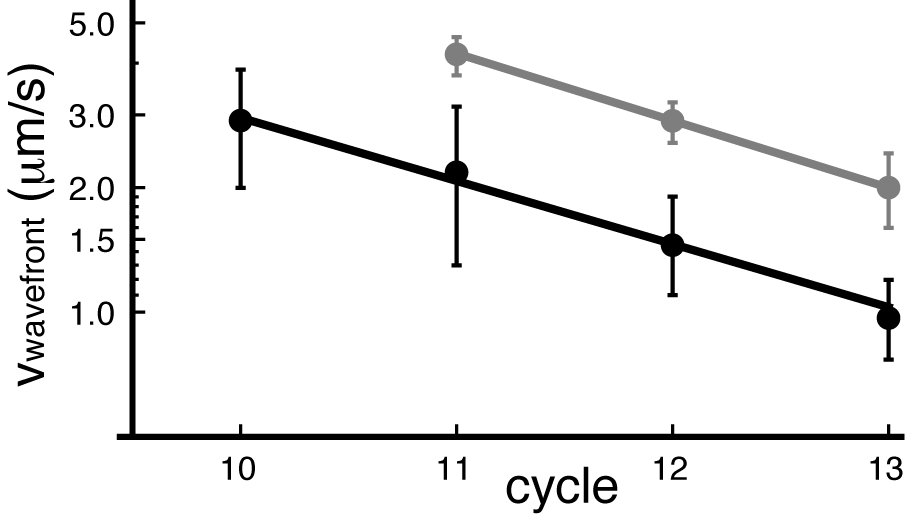

Supplement: Figure S3 — Average speed of each of the two sets of data, on a log-linear plot. The data are fitted by an exponential , . The black dots correspond to the mean wavefront speeds of set 1, and the gray ones to the mean speeds of set 2. (TIFF) [file pone.0077216.s003.tiff]

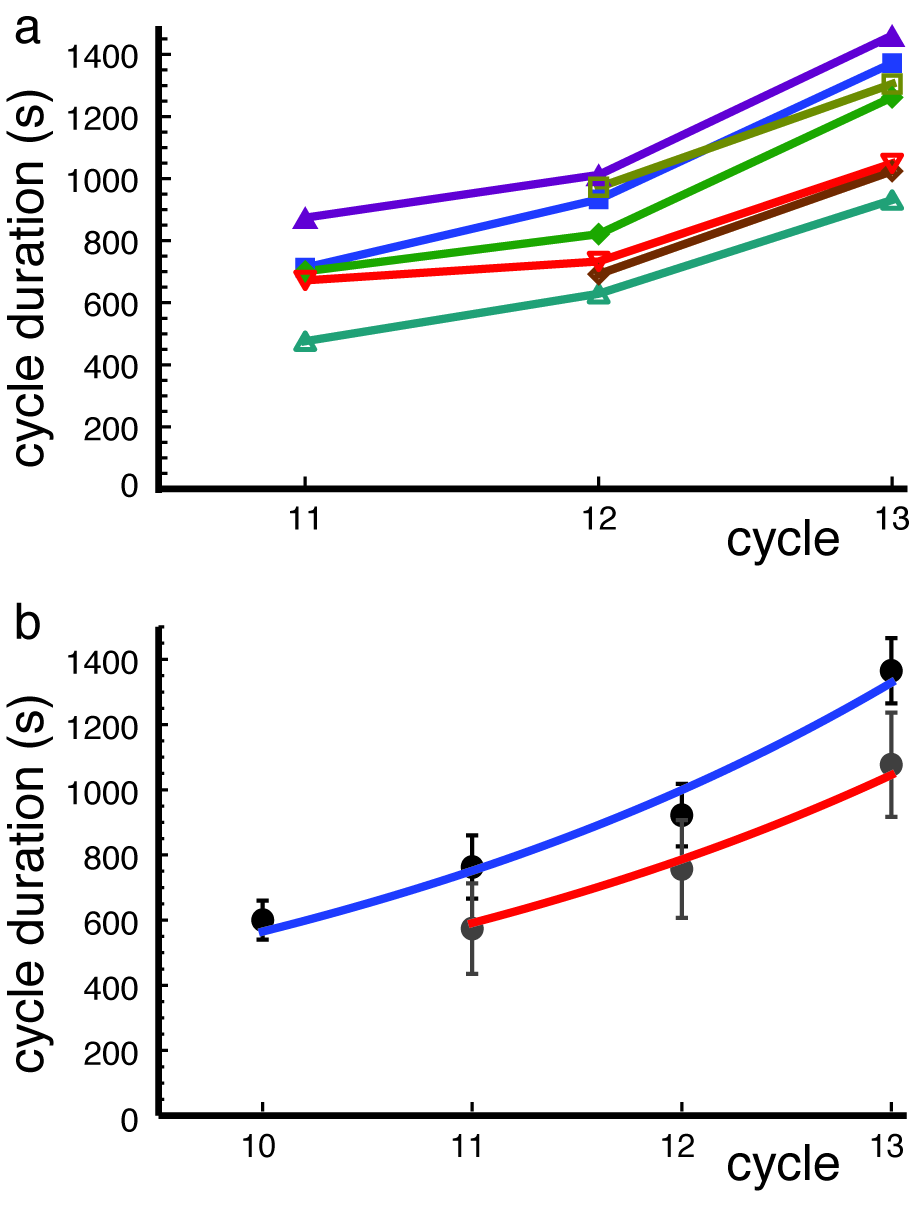

Supplement: Figure S4 — Duration of the measured cycles. a) Experimental data. The different symbols and colors correspond to the ones in Figure 2. b) Cycle duration averaged over all experimentally observed embryos (black and gray dots for sets 1 and 2 respectively). The cycle durations can be fitted reasonably well by a weak exponential , where (set 1) and (set 2). (TIFF) [file pone.0077216.s004.tiff]
