# Supplementary material for: The Syncytial Drosophila Embryo as a Mechanically Excitable Medium
Source: PLoS One. 2013 Oct 30;8(10):e77216. doi: 10.1371/journal.pone.0077216 (PMC3813724; doi:10.1371/journal.pone.0077216)
Supplement: File S1 — Supplementary information, in which we discuss the various models in more detail: (1) Timing models for wavefront propagation; (2) Diffusion model for wavefront propagation, including models with time delay; and (3) Mechanical model for wavefront propagation. We also analyze the steady-state of a propagating wavefront in both the diffusion and mechanical model. (PDF) [file pone.0077216.s005.pdf]

Supplement to The syncytial *Drosophila* embryo as a mechanically excitable medium

Timon Idema, Julien O. Dubuis, Louis Kang, M. Lisa Manning, Philip C. Nelson, Tom C. Lubenksy, and Andrea J. Liu

October 2, 2013

1 Timing models

Consider the following timing mechanism for generating a wavefront in a row of people. Assume each person has a (synchronized) watch, and each is told to raise his/her arms at exactly τ seconds, according to their individual clocks, after an arbitrarily chosen $t = 0$. If all of the clocks run at the same rate, there is no wave, but if the clock of each successive person in the line runs more slowly that that of his/her neighbor to the right, then a wavefront will be generated.

For the timing method to be the cause for the wavefronts observed in our system, each nucleus would require a clock. That clock could simply be the amount of time it takes to duplicate the DNA, i.e., the duration of the interphase, which changes from one cycle to the next. However, there is no correlation between a nucleus' position and the duration of interphase, which means that we would not expect a mitotic wavefront to emerge in this case. Alternatively, it is well-established that there are several proteins which exhibit patterning along the anterior-posterior or dorsal-ventral axes of the embryo. A much studied example is Bicoid, which exhibits an exponential profile along the anterior-posterior axis [1,2], the same axis along which the wavefront travels. Now if the duration of interphase were affected by the local Bicoid concentration, that could provide a mechanism for the clocks of the nuclei to get out of sync, and produce a wavefront in the various markers for mitosis (such as our observed metaphase and anaphase wavefronts).

There are three reasons why the model outlined above cannot explain our data. The first is specific to Bicoid. As observed by Gregor et al. [2], the total amount of Bicoid steadily increases over time, as more of the protein is translated in each cycle. In particular, the amount of protein keeps steady pace with the number of nuclei, such that at the start of each cycle, the actual amount of protein in each nucleus at a given position in the embryo is always the same. Therefore if Bicoid were responsible for causing the mitotic wavefronts, the wavefronts would have the same speed in each cycle, which they do not. The second reason is more general: as we show below, in order to obtain a linear wavefront propagation from an exponential concentration profile, the actual absolute amount of material does not matter,

only the decay length - which means that once again the predicted wavefront speed would be independent of cycle. Finally note that to get wavefronts traveling in both directions along the anterior-posterior axis of the embryo, we would need at least two concentration gradients of different proteins, for which it would be highly surprising if they produced wavefronts with the same speed. The timing method therefore cannot describe our data.

We now assume that the nuclei do *not* signal each other, in any way, that it is time to start mitosis; instead, they sample their local environment for a given protein, such as Bicoid (Bcd), and have the length of their cycle depend on the local concentration. Because the concentration of many such proteins does indeed exhibit a gradient from one of the two poles, this could explain how mitosis starts close to the poles, and then seems to 'travel' along the embryo, where in reality there is no traveling front at all.

Roughly speaking, there are four kinds of patterns expressed by proteins in the early *Drosophila* embryo: an exponential profile along the AP axis starting from one of the poles (of which Bcd is an example [1,2]), an exponential profile along the DV axis (such as Dorsal [3]), a terminal morphogen profile which is high at both poles and both low and flat in between (as for the phosphorelation gradient of MAPK [3]), and a striped pattern (for e.g. Hunchback, Giant, Paired and Runt [4]). Because the observed mitotic waves start at the poles and then spread along the AP axis of the embryo, the striped patterns and the DV-axis gradients cannot be the ones causing them. There are often *two* mitotic waves, which start at the two opposite poles, which suggest that the terminal morphogens might be good candidates, but they hardly show any profile in the mid-60% of the embryo [3]. The most likely candidates are therefore the proteins that have an exponential profile along the AP axis, although in this case there must be at least two proteins that can trigger mitosis. This latter observation is a first weak point of the timing model, but not necessarily cause to rule it out.

During interphase, the nuclei in the syncytial embryo are surrounded by a nuclear membrane (also known as the nuclear envelope). One of the things the nucleus can do is to concentrate proteins inside that membrane. This has been observed directly for Bcd by Gregor et al. [2] and confirmed by our own observations. Irrespective of whether the proteins are concentrated in the nuclei during interphase, or they are distributed throughout the entire cytosol, they always exhibit an exponential decay along the anterior-posterior axis. As observed by Gregor et al. [2], the total amount of Bcd steadily increases over time, as more of the protein is translated in each cycle. In particular, the amount of protein keeps steady pace with the number of nuclei, such that at the start of each cycle, the actual amount of protein in each nucleus at a given position in the embryo is always the same [2].

Let us denote the position along the long axis by x, and the total length of the axis by L. The local concentration at x is then given by $c(x) = c_0 e^{-x/\lambda}$, where λ is the characteristic length scale of the exponential profile. As stated above, the experimental results of Gregor et al. tell us that λ is the same in all cycles, whereas c_0 goes up [2]. Assuming that all nuclei are equally good at collecting material from their environment, the amount of material collected by a single nucleus in a simple one-dimensional model of the embryo is given by

$$C(x,N) = \int_{x-L/2N}^{x+L/2N} c(y)\,\mathrm{d}y = 2c_0\lambda \sinh\left(\frac{L}{2N\lambda}\right) e^{-x/\lambda}, \tag{1}$$

where N is the number of nuclei. As stated above, the key assumption of the timing model is that the duration of the cycle of each nucleus depends somehow on the concentration, or rather, on the amount of material in the nucleus, so we have

$$\Delta t_{\text{cycle}} = f(C(x,N)) = f\left(2c_0\lambda \sinh\left(\frac{L}{2N\lambda}\right) e^{-x/\lambda}\right). \tag{2}$$

Unfortunately, we do not know what the function f in equation (2) is. The only thing we do know is that it is monotonously increasing with its argument (the total amount of material in a nucleus). We will therefore explore two explicit possibilities:

- The simplest possible dependence: f is a linear function.
- The dependence that gives the observed behavior of (effective) wavefronts, i.e., that the resulting 'speed' v of mitosis events through the embryo is well-defined and constant throughout, and $\Delta t_{\text{cycle}} = x/v$.

For the first option, we write $f(\alpha) = t_0 - \tau\alpha$, with t_0 some offset time and τ a timescale. We can then calculate the speed of an observed mitotic wavefront, as a function of the number of nuclei N, by calculating the time difference between two positions x and y in the embryo:

$$\begin{aligned} v(x,y,N) &= \frac{y-x}{\Delta t_{\text{cycle}}(y,N) - \Delta t_{\text{cycle}}(x,N)} \\ &= \frac{y-x}{2c_0\lambda\tau \sinh\left(\frac{L}{2N\lambda}\right)\left(e^{-x/\lambda} - e^{-y/\lambda}\right)}. \end{aligned} \tag{3}$$

Equation (3) shows that v depends on the position, so there is no well-defined wavespeed in this model. This is of course no big surprise - we just took a random functional dependence for f, so there should be no reason to expect it would produce a wavespeed that is position-independent. However, this does illustrate the point that a constant wavespeed is something special: we need to specifically *choose* f such that a constant speed comes out.

Note that it may of course be that there is no constant wavespeed, but that it only appears to be constant within our error bars. Although we can not rule this option out, this also does not come out naturally. For instance, inserting numbers for *Drosophila* from Gregor et al. [4] ($L = 450\mu$m, $\lambda = 70\mu$m $= 0.15L$, $N = 50$) we find that for $x = 0$, the measured speed more than doubles as we take the measuring point y across the embryo, which can certainly not be confused with a constant speed.

To get a constant speed, we need to choose a different function f, specifically a logarithm: $f(\alpha) = t_0 + \tau \log \alpha$. In this case we find:

$$v(x,y,N) = \frac{y-x}{(\tau/\lambda)(y-x)} = \lambda/\tau. \tag{4}$$

In this case, we do indeed find a constant value of v across the embryo. However, we also find that v is independent of N. v does depend on the decay length λ, but the value of λ does not change [2]. The wavefront speed predicted by this model is therefore the same for all cycles, in direct contradiction to our observations.

2 Diffusion model

The process of diffusion is governed by the diffusion equation, here given for a concentration field $c(\vec{x}, t)$:

$$\frac{\partial c(\vec{x}, t)}{\partial t} = D \nabla^2 c(\vec{x}, t), \tag{5}$$

where D is the diffusion constant. Equation (5) is linear, so we can use the superposition principle: the sum of any two solutions is itself a solution. The general solution for a system with no boundaries depends only on the initial condition $c(\vec{x}, 0)$, and is given by

$$c(\vec{x}, t) = \int G(\vec{x}, \vec{y}, t) c(\vec{y}, 0) \, \mathrm{d}\vec{y}, \tag{6}$$

where $G(\vec{x}, \vec{y}, t)$ is the Green's function of the diffusion equation, which for a system in n dimensions is given by

$$G(\vec{x}, \vec{y}, t) = \frac{1}{(4\pi D t)^{n/2}} \exp\left(-\frac{|\vec{x} - \vec{y}|^2}{4Dt} \right). \tag{7}$$

The Green's function describes the concentration field at $\vec{x}$ at time t due to a single delta-function concentration source at $\vec{y}$ at time 0.

3 Mechanical model

As described in the main text, we can describe the medium in which the nuclei live as an overdamped elastic medium. Motion in this medium can be described by some displacement vector $\vec{u}$ from a fixed reference position. We get force balance by equating the damping forces acting on the nuclei (due to friction with the cortical actin layer surrounding the yolk or the outer membrane, and drag due to the viscous fluids the nuclei and their surrounding microtubule baskets are immersed in) to the elastic forces in the polymer cytoskeleton:

$$\Gamma \partial_t u_i = \frac{E}{2(1+\nu)} \partial_j \partial_j u_i + \frac{E}{2(1-\nu)} \partial_i \partial_j u_j. \tag{8}$$

As also pointed out in the main text, equation (8) is reminiscent of the diffusion equation: a time derivative on the left equals second-order space derivatives plus a source term on the right, and it comes as no surprise that the solution depends on a quantity with the dimension of a diffusion constant $D = \mu/\Gamma$, where μ is the material's shear modulus and equals $E/(2+2\nu)$. Moreover, equation (8) also allows for a Green's function type solution, but here in the form of a tensor $G_{ijk}(\vec{x}, t)$, relating an arbitrary input $Q_{ij}\delta(\vec{x})\Theta(t)$ to a resulting displacement vector $u_k(\vec{x}, t)$ [5]. In two dimensions, the input tensor Q_{ij} has three independent components, and can be decomposed in a (hydrostatic) expansion/contraction, and two symmetric traceless parts:

$$\mathbf{Q} = Q_0 \begin{pmatrix} 1 & 0 \\ 0 & 1 \end{pmatrix} + Q_1 \begin{pmatrix} 1 & 0 \\ 0 & -1 \end{pmatrix} + Q_2 \begin{pmatrix} 0 & 1 \\ 1 & 0 \end{pmatrix}. \tag{9}$$

The two symmetric traceless parts can be converted into each other by a rotation over $\pi/4$. They can therefore alternatively be written as a single term with a prefactor and an angle, as done in equation (3) of the main text:

$$\mathbf{Q} = Q_0 \begin{pmatrix} 1 & 0 \\ 0 & 1 \end{pmatrix} - Q_1 \begin{pmatrix} \cos 2\theta & \sin 2\theta \\ \sin 2\theta & -\cos 2\theta \end{pmatrix}. \tag{10}$$

The second term in equation (10) now represents a volume-conserving force dipole that is oriented at an angle θ with respect to the x-axis. An example displacement field due to a single force dipole at the origin is given in Figure S5.

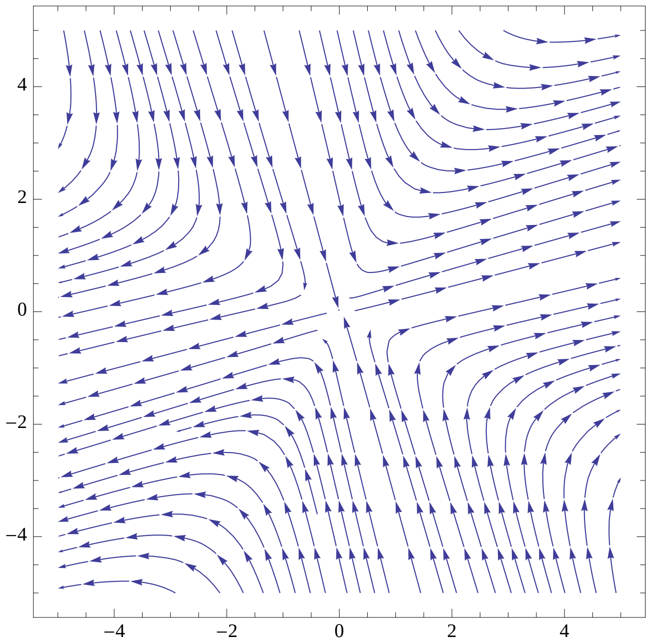

Figure S5. Example of a displacement field due to a single force dipole located at the origin and having an angle of $\pi/6$ with respect to the x-axis, obtained by taking the $t \to \infty$ limit of $G_{ijk}(\vec{x}, t)Q_{ij}$.

4 Wavefronts

Now that we know the solutions to the chemical and mechanical diffusion equations due to a single source, we can exploit the fact that the chemical and stress diffusion equations (5 and 8) are linear to compute the behavior of a system with many sources using the superposition principle. For simplicity, we pre-arrange the nuclei on a triangular grid, with a little noise in the position of each nucleus to prevent artifacts due to a perfect arrangement. This is consistent with our observation that just before the mitosis waves the nuclei in an actual embryo have a rather high degree of triangular order, except where there are defects due

to the fact that a nucleus did not divide in an earlier cycle. Alternatively, we can also consider packings with short-range correlations but no long-range order (like the packing of soft repulsive spheres), which gives the same results [5].

4.1 Wavefronts in the biochemical diffusion model

We will describe the release by a nucleus of a biochemical with a Dirac delta function source located at the position of the nucleus. Because integration is a linear operation, adding two sources, even if they divide at different times, is trivial - we simply carry out the integration in equation (6) for each nucleus that has already divided with the time properly offset, then sum over these nuclei. The only problem is to determine when each nucleus is supposed to divide. To find out, we perform what is essentially a numerical integration over time. We start with a release of material at the origin, which we model by having a delta function concentration there at $t = 0$. By construction, the concentration field is then given by $G(\vec{x}, 0, t)$ as long as no other sources have released their chemicals into the system. We proceed in small timesteps Δt, calculating for each timestep the concentration at the location of each of the nuclei that have not yet divided, given the total concentration field generated by the nuclei that have divided so far. Suppose there is a total number of N nuclei, M of which have already released their chemicals. The ith source is located at $\vec{x} = \vec{a}_i$, released its chemicals at $t = t_i$, and the t_i's are ordered. For $t_M < t < t_{M+1}$ we then find by using the superposition principle:

$$c(\vec{x}, t) = \sum_{i=1}^{M} \frac{Q}{4\pi D(t - t_i)} \exp\left(-\frac{|\vec{x} - \vec{a}_i|^2}{4Dt}\right), \tag{11}$$

where we have taken the number of dimensions to be two, and Q is the number of chemicals released by a single nucleus. From $c(\vec{x}, t)$ we can determine when the next source will release its chemicals, by solving $c(\vec{a}_j, t) = \alpha$ for $M + 1 \leq j \leq N$. We thus check all nuclei that have not yet released anything, and determine which one will be the next source by finding the one with the smallest value of t, which sets t_{M+1}. If there is a nonzero delay time between activation and release, we simply add it to the found value of t_{M+1}. Given the positions of the sources, the system thus has three parameters: the diffusion constant D, the release concentration α, and the delay time t_{delay}. The 'release-wave' is then the time at which a given source releases its chemicals versus its distance to the origin (i.e., the first source). An example is given in Figure 3a of the main text, which reveals a clear wavefront with a well-defined wave speed.

4.1.1 Algorithm for finding wavefronts

In summary, we use the following algorithm to numerically find the wavefronts within the biochemical diffusion model (and, with the proper adaptations, for the stress diffusion model as well):

- Generate a grid of hexagonally arranged nuclei with some small positional noise, centered at the origin.

- Start with a delta function concentration at the origin at $t = 0$.
- Increase time in steps of Δt. For each timestep, calculate the local concentration at each of the sites of the nuclei, due to the nuclei that have released chemical so far. If one of these exceeds the critical concentration α, add a delta function concentration peak at this location and time.
- Stop after either all nuclei have divided or a predefined time has been reached.

4.1.2 Analysis of the steady-state wavefront

In the case without delay time, it is fairly easy to determine the speed of the wavefront for the regime in which the wavefront is well-established, i.e., when its curvature is small. Suppose the lattice spacing is a, the time it takes to get from one row to the next is t, and the amount of material released by each nucleus is Q. The speed, in a triangular lattice, is then $v = a \cdot \frac{1}{2}\sqrt{3}/t$, because the spacing between the two rows is $a \cdot \frac{1}{2}\sqrt{3}$. To find the time, it turns out to be sufficient to only consider the 2 nearest neighbors in the previous row. We choose coordinates such that the neighbors are located at $(\pm\frac{a}{2}, 0)$, and our next nucleus is at $(0, \frac{a}{2}\sqrt{3})$. Then the time at which this next nucleus is activated is given by the solution of

$$\alpha = \frac{2Q}{4\pi Dt} \exp\left[-\frac{a^2}{4Dt}\right]. \tag{12}$$

The results obtained using equation (12) are almost identical to those obtained by numerical solution of the full equations. If necessary, corrections could of course be made by including additional sources.

To analyze equation (12) further, we introduce dimensionless variables $\bar{\alpha} = a^2\alpha/Q$ and $\tau = Dt/a^2$. The equation then becomes

$$\bar{\alpha} = \frac{1}{2\pi\tau} e^{-1/4\tau}, \tag{13}$$

which can of course not be solved analytically, but is easy to solve numerically. We note that the right hand side of (13) has a maximum value of $\bar{\alpha}_{\max} = 2/\pi e$ at $\tau = 1/4$, giving the condition that there can only be a wave if $\alpha < 2Q/\pi e a^2$. If we write the inverse of (13) as $\tau = f(\bar{\alpha})$, we can write for the wavefront speed

$$v = \frac{D}{a}\frac{1}{2}\sqrt{3}\frac{1}{f(\bar{\alpha})}, \tag{14}$$

so $v \propto D$ if both a and α are fixed, but unfortunately the scaling of the speed with a and α is hidden in $f(\bar{\alpha})$. Based on the numerical determination of $f(\bar{\alpha})$ we can capture its features fairly accurately with the following function

$$f(\bar{\alpha}) \approx b_1 \bar{\alpha}^{1/n} \left[\frac{1}{4} - b_2\left(\frac{8}{e} - \bar{\alpha}\right)^{1/m}\right], \tag{15}$$

where fitting gives $b_1 = 1.38$, $b_2 = 0.28$, $n = 6.3$ and $m = 3.6$ (see Figure S6). Our numerical solutions of the full equations show that the data do indeed collapse onto the curve described by equations (14) and (15) for different diffusion coefficients and nuclear spacings (Figure S7). In particular, we find that the wavefront speed v always *increases* as the nuclear spacing a *decreases*, so v increases with increasing cycle number.

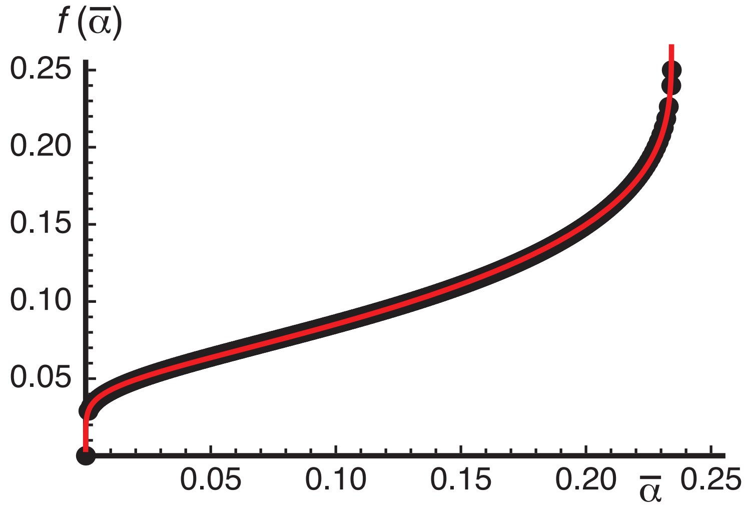

Figure S6. Plot of $f(\bar{\alpha})$, defined in equation (14), determined numerically (black dots) and fitted with equation (15) (red line).

4.2 Wavefronts in the biochemical diffusion model with delay time

As indicated in Section 4.1, we can include a delay time t_{delay} into our diffusion model. Now a nucleus divides (i.e., releases its chemicals) a time t_{delay} *after* the local concentration first reaches the threshold value α. To first approximation, the total time between the activation of a nucleus in a given row and one in the next row is simply the sum of the delay time and the travel time, which we found in the previous section. We therefore find:

$$v = \frac{Da\sqrt{3}}{2Dt_{\text{delay}} + 2a^2 f(a^2\alpha/Q)}. \tag{16}$$

Equation 16 breaks down for long delay times, or small thresholds, as in those cases the effects of earlier releases become important. However, for our system these effects are small. Figure S8 shows the wavefront speed v as a function of cycle number for four different values of the delay time, and for both the cases that α and $\bar{\alpha}$ are constant. Note that for constant α there is a lower limit to the cycle number below which the model predicts no wavefronts (as the activation threshold is not reached). Note also that although the introduction of the delay time causes the increasing trend in the wavefront speed to chance in the later cycles, it will still increase in earlier cycles.

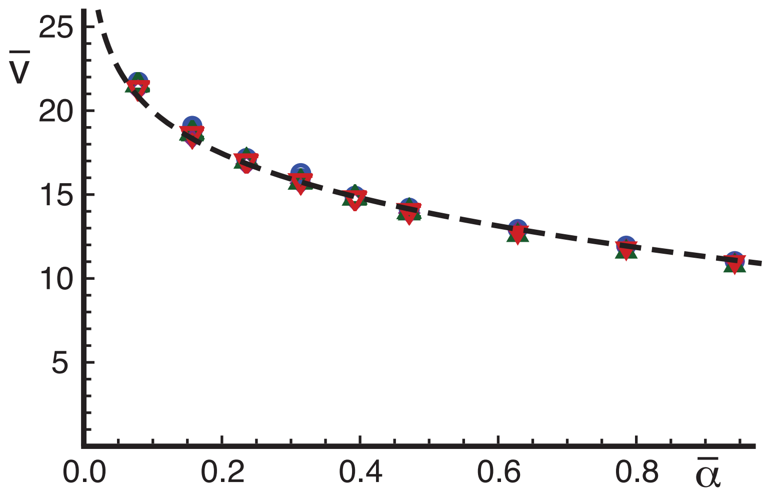

Figure S7. Dimensionless wavefront speed $\bar{v} = v/(D/a)$ as a function of the dimensionless threshold $\bar{\alpha} = a^2\alpha/Q$ in the biochemical signaling model. The figure shows $\bar{v}$ for several choices of the parameters D and a (different symbols). The data all collapse on a single curve, as described by equation (14). The dashed line is the fit to that curve of equation (15).

4.2.1 Fitting the data with time delay

Naturally, the more parameters we have, the easier it is to fit any set of experimental data points. In the diffusion model with delay time we have four parameters: the diffusion constant D, the grid size a, the threshold value α and the delay time t_{delay}. The grid size (i.e. the spacing between the nuclei) is measured independently, leaving us with three parameters which we can vary. Reasonable values for the diffusion constant for a small chemical in the early *Drosophila* embryo are $D = 5 - 100$ $\mu\text{m}^2/\text{s}$, as measured by Gregor et al. [4]. As indicated in the main text, we cannot fit the experimental trend (a wavefront speed that decreases exponentially with cycle number) with fixed values for t_{delay} and α for any value of D within this range (see Figure 3b of the main document). The only way we can thus fit the experimental data within this model is if either (or both) of α and t_{delay} change with the cycle number.

We have systematically investigated a number of options, changing α or t_{delay} with cycle number. Some results are given in Table S1. We did not find any result that fit the data in which the numbers change in a well-defined way (e.g. the delay time increasing linearly or exponentially with the cycle number). Moreover, in the case of variable delay time, we find that in the first cycle (cycle 10), the interaction is between nearest-neighbors as in the model without time delay, but the interaction range goes up every cycle, up to 5 rows apart in cycle 13. In the case of variable concentration threshold, we need a change of at least an order of magnitude in each cycle to fit the experimental data. Even in the case where we allow both variables to change, we keep finding at least one of these two problems. Even though

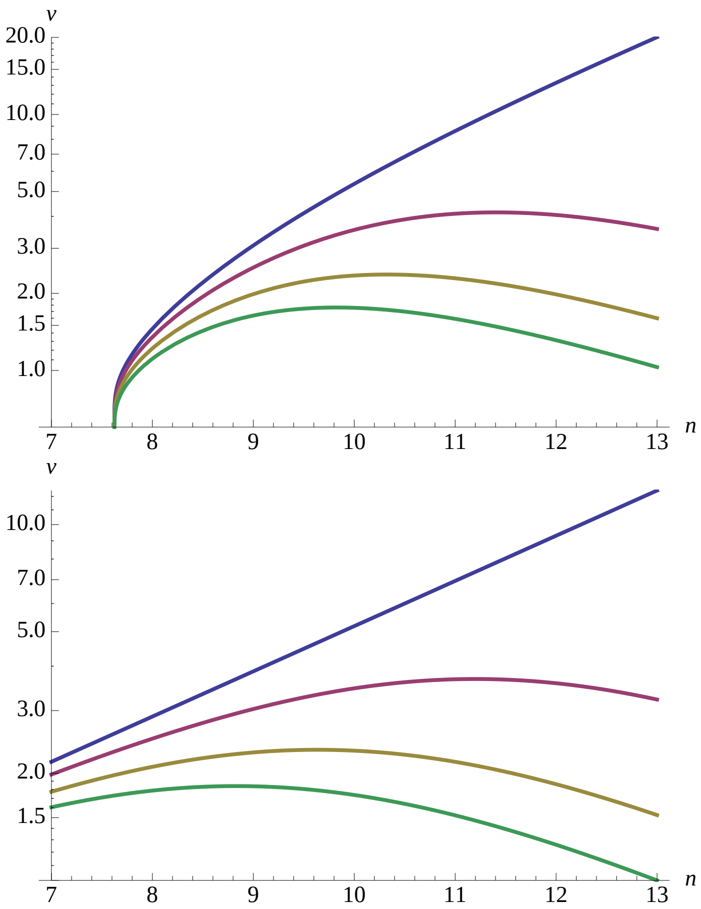

Figure S8. Log-linear plots of the wavefront speed as a function of cycle number in the diffusion model with time delay. Top: fixed threshold $\alpha/Q = 0.001\mu\text{m}^{-2}$, $D = 10\mu\text{m}^2/\text{s}$, and four values of t_{delay}: 0s (blue), 2s (red), 5s (gold), and 8s (green). Bottom: same graph for fixed rescaled threshold $\bar{\alpha} = 0.06$.

we cannot strictly rule out the diffusion model with variable time delay and concentration threshold, these results make it very unlikely that this model is actually correct.

4.3 Wavefronts in the mechanical model

The analysis leading to wavefront propagation in the mechanical model is described in Ref. [5]. As in the case of diffusion, we must set a threshold to determine when a source (a nucleus) is activated. The simplest option is to look at the eigenvalues of the stress tensor: if the largest of those (taking absolute values) exceeds a certain threshold α, the nucleus is activated. An activated nucleus adds an additional force dipole term to the stress field in the system, which in turn of course affects the displacement field, as described by the Green tensor solution of equation (8). Note that we assume linear elasticity, so that the strain is

linear in the displacement, and the stress is linear in the strain. The superposition principle therefore holds not just for the displacements but for the strain and stress fields as well.

The implementation of the stress-mediated signaling model follows the same pattern as that of the chemical-diffusion-mediated signaling model, with the concentration c replaced by the stress tensor σ_{ij}. An example implementation on a grid of 21×21 nuclei is shown in Figure S9. The figure shows a clear wavefront which has a well-defined speed.

As in the diffusion model, we analyze our mechanical model in terms of dimensionless parameters. There is only one quantity in our model that has the dimensions of a speed, namely $\mu/a\Gamma$, which means that the resulting wavefront speed has to scale linearly with this factor, as indeed it does. We again define a dimensionless wavefront speed $\bar{v} = \frac{a\Gamma}{\mu} v = \frac{v}{D/a}$ and a dimensionless stress threshold $\bar{\alpha} = a^2\alpha/Q$, where Q is now the strength of the force dipole. We can then write

$$v = \frac{\mu}{a\Gamma} g(\bar{\alpha}, \nu) \tag{17}$$

We determine the function $g(\bar{\alpha}, \nu)$ by numerically solving the model. As detailed in [5], we find that it can be well described by the following functional form, derived using a similar argument we used to arrive at equation (13) for the diffusion model:

$$g(\bar{\alpha}, \nu) = -4\frac{(c_1 + c_2\bar{\alpha})\log(\bar{\alpha})}{1 - \nu^2} + c_3. \tag{18}$$

Note that the form in equation (18) differs slightly from that in [5] because of the use of the shear modulus μ instead of the Young's modulus E in the definition of $\bar{v}$, and the extra constant c_3, which is due to the introduction of semi-periodic boundary conditions. We use equations (17) and (18) with $c_1 = 4.5$, $c_2 = 1.5$ and $c_3 = 5.4$ to fit the experimental data in figure 3f of the main text.

References

[1] Driever W, Nüsselein-Volhard C (1988) A gradient of *bicoid* protein in *Drosophila* embryos. Cell 54: 83-93.

[2] Gregor T, Wieschaus EF, McGregor AP, Bialek W, Tank DW (2007) Stability and nuclear dynamics of the bicoid morphogen gradient. Cell 130: 141-152.

[3] Sample C, Shvartsman SY (2010) Multiscale modeling of diffusion in the early *Drosophila* embryo. Proc Natl Acad Sci U S A 107: 10092-10096.

[4] Gregor T, Bialek W, de Ruyter van Steveninck RR, Tank DW, Wieschaus EF (2005) Diffusion and scaling during early embryonic pattern formation. Proc Natl Acad Sci U S A 102: 18403-18407.

[5] Idema T, Liu AJ (2013) Mechanical signaling via nonlinear wavefront propagation in a mechanically-excitable medium. arXiv:1304.3657.

cycle number	nuclear spacing (μm)	wavefront speed (μm/s)	t_{delay} (s) (α fixed)	α/Q ($10^{-4}/\mu\mathrm{m}^2$) (t_{delay} fixed)	(t_{delay}, α/Q) (s, $10^{-4}/\mu\mathrm{m}^2$)
10	23.4 ± 0.8	2.9 ± 0.9	1.5	0.0044	(5, 0.254)
11	18.2 ± 0.6	2.2 ± 0.9	5.7	0.29	(10, 0.300)
12	13.2 ± 0.3	1.5 ± 0.4	16.0	6.9	(20, 0.685)
13	9.7 ± 0.2	1.0 ± 0.2	36.1	31	(40, 1.057)

Table S1. Experimentally determined values of the nuclear spacing and wavefront speed (set 1), and numerically determined values of the required delay time t_{delay} and threshold concentration α to fit the experimental data. In column four, $D = 15\ \mu\mathrm{m}^2/\mathrm{s}$ and $\alpha/Q = 5 \cdot 10^{-4}\ \mu\mathrm{m}^{-2}$; in column 5, $D = 10\ \mu\mathrm{m}^2/\mathrm{s}$ and $t_{\mathrm{delay}} = 10$ s; in column 6, $D = 10\ \mu\mathrm{m}^2/\mathrm{s}$ and t_{delay} is assumed to double in each cycle. None of the columns show a systematic dependency of the parameters on the cycle number, making it impossible to assign predictive power to the numbers found, or to find a model to explain the dependencies. Note also that for the case of fixed delay time (column 5), we need to assume that the threshold value goes up by at least an order of magnitude in each cycle. In the case of variable delay time (columns 4 and 6), the interactions become very long-ranged in the later cycles, with nuclei up to 5 rows apart triggering each other in cycle 13, even though in cycle 10 the interaction only involves nearest neighbors.

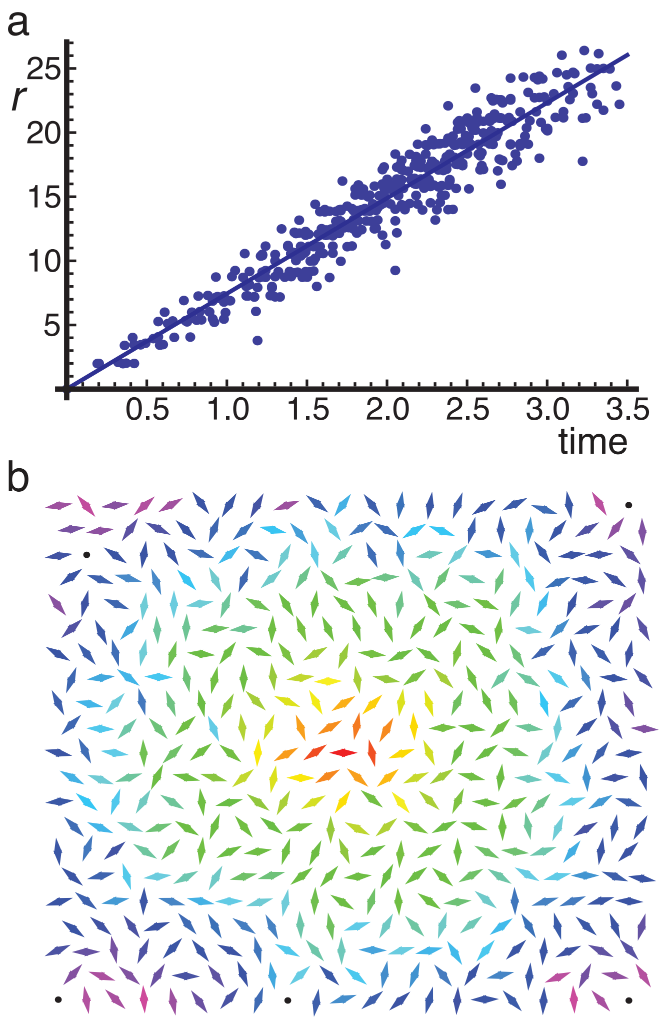

Figure S9. Wavefront from a simulation with 21×21 nuclei. The nuclei are arranged on a hexagonal grid with random small offsets. The wave starts at the center point which generates a stress dipole of unit strength along the x-axis at $t = 0$. Whenever the absolute value of the largest eigenvalue of the stress tensor at another nucleus exceeds the threshold value α, it also divides, adding a unit stress dipole in a random direction to the total stress field. a) Distance of the dividing nuclei to the center vs. their activation time, with a linear fit. b) Graphical representation of the 2 dimensional field, with the dipoles indicated in the orientation in which they divide, and color-coded according to the time that they divide, on a hue scale (red-yellow-green-blue-violet).
